# SARS-CoV2 Infection Alters Tryptophan Catabolism and Phospholipid Metabolism

**DOI:** 10.3390/metabo11100659

**Published:** 2021-09-28

**Authors:** Gagandeep Kaur, Xiangming Ji, Irfan Rahman

**Affiliations:** 1 Department of Environmental Medicine, University of Rochester Medical Center, Rochester, NY 14642, USA; Gagandeep_Kaur@URMC.Rochester.edu; 2 Department of Nutrition, Byrdine F. Lewis School of Nursing and Health Professions, Georgia State University, Atlanta, GA 30302, USA; xji4@gsu.edu

**Keywords:** metabolomic, SARS-CoV2, COVID-19, tryptophan metabolism, lipid metabolism

## Abstract

Coronavirus disease 2019 (COVID-19) has so far infected hundreds of million individuals, with several million deaths worldwide. The lack of understanding of the disease pathophysiology and the host’s immune response has resulted in this rapid spread of the disease on a global scale. In this respect, we employed UPLC-MS to compare the metabolites in the serum from COVID-19-positive patients and COVID-19-recovered subjects to determine the metabolic changes responsible for an infection. Our investigations revealed significant increase in the levels of serum phospholipids including sphingomyelins, phosphatidylcholines and arachidonic acid in the serum of COVID-19-positive patients as compared to COVID-19-recovered individuals. We further show increased levels of tryptophan and its metabolites in the serum of COVID-19-positive patients thus emphasizing the role of tryptophan metabolism in the disease pathogenesis of COVID-19. Future studies are required to determine the changes in the lipid and tryptophan metabolism at various stages of COVID-19 disease development, progression and recovery to better understand the host–pathogen interaction and the long-term effects of a severe acute respiratory syndrome coronavirus 2 (SARS-CoV2) infection in humans.

## 1. Introduction

The year 2020 was marked with the spread of the global pandemic of Coronavirus disease (COVID-19) caused by severe acute respiratory syndrome coronavirus 2 (SARS-CoV2). As per the World Health Organization (WHO) estimate, approximately 3 million global “excess deaths” (deaths beyond the normal limits) could be attributed to COVID-19 infection, either directly or indirectly [1]. While the disease presents with silent or mild symptoms in most patients, it can develop into pneumonia that may progress to respiratory failure within 7–10 days’ time after the first symptoms [2,3]. Emergence of newer variants with higher transmissibility adds to the challenge of infection containment [4,5]. Furthermore, there are reports of long-term complications due to lung remodeling (fibrosis) in some patients after recovery. This makes it important to understand the disease pathogenesis and identify biomarkers that could predict disease severity for better management.

In this respect, various ‘omic’ techniques have been used by multiple groups, identifying potential targets/pathways affected by SARS-CoV2 infection [6,7,8,9]. Indeed, there have been several metabolomics studies conducted so far that identify the dysregulated pathways in COVID-19, but further work in this area is important to draw meaningful conclusions regarding the disease pathogenesis in COVID-19 [2,3,8,10,11,12,13,14].

Considering this, we compared the metabolomic profiles of serum samples from COVID-19-positive and COVID-19-recovered subject groups to identify the metabolites and potential metabolic pathways that were dysregulated on SARS-CoV2 infection. Our general hypothesis was that the systemic metabolic signatures of existing patients with a COVID-19 infection are distinct from those in COVID-19-recovered individuals, and identifying these metabolic variations will provide insight into the disease’s development and progression. 

## 2. Results

### 2.1. Mass Spectra in Positive and Negative Ion Mode of Serum from COVID-19-Positive and COVID-19-Recovered Subjects Analyzed by UPLC-MS

**Figure 1:**
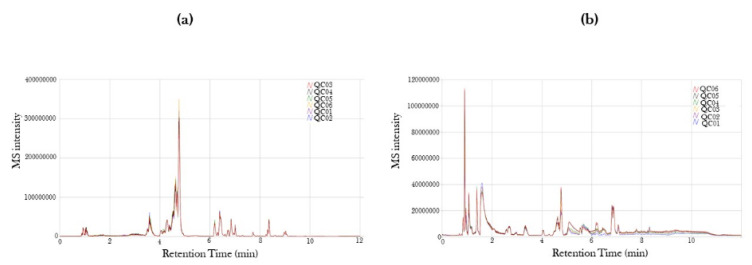
Mass spectra of retention time version ion intensity for COVID-19-positive and COVID-19-recovered patient serum samples. Serum samples from COVID-19-positive and COVID-19-recovered patients were obtained and the serum metabolites were characterized using ultra-performance liquid chromatography mass spectrometry (UPLC-MS). Mass Spectra from (**a**) positive and (**b**) negative ion modes are represented as the initial quality check to identify individual metabolites.

The altered metabolite profiling of the serum samples from COVID-19-positive and COVID-19-recovered subject groups was performed in positive and negative ion modes by UPLC-MS. The mass spectra of MS intensity (Y-axis) versus retention time (X-axis) was obtained for all the identified metabolites in positive and negative ion mode (Figure 1). We identified 3788 and 1725 metabolite peaks in positive and negative ion modes, respectively, in our serum samples.

**Figure 2:**
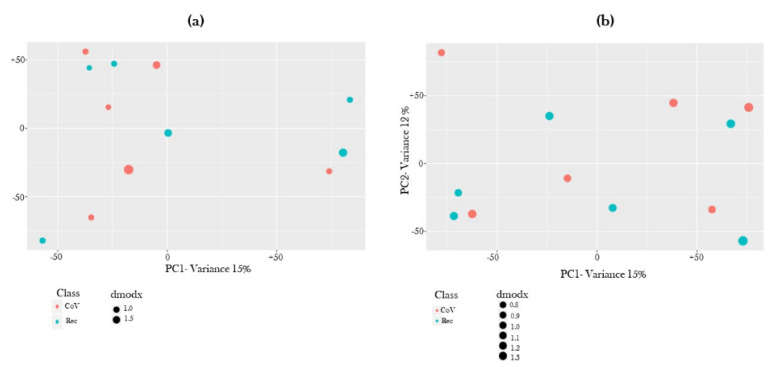
Principle component plot for COVID-19-positive and COVID-19-recovered patient serum samples. Principle component plot of (**a**) positive and (**b**) negative ion modes based on differential levels of metabolites in the serum samples from COVID-19-positive and COVID-19-recovered patients as quantified using ultra-performance liquid chromatography mass spectrometry (UPLC-MS).

Principal Component Analysis of the identified metabolites in each serum sample was plotted for both ion modes (Figure 2). As expected, we observed overlapped metabolite distribution in COVID-19-positive and COVID-19-recovered subject groups. 

### 2.2. Global Metabolite Profiling Identified Significantly Dysregulated Metabolites in COVID-19-Positive Patients as Compared to COVID-19-Recovered Subjects

**Figure 3:**
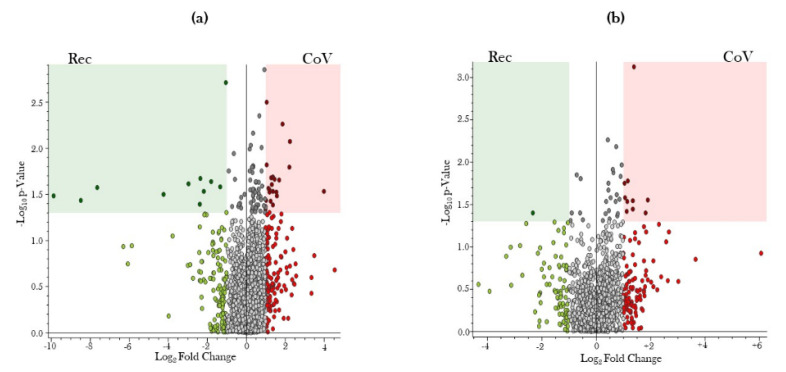
Volcano plot showing number and distribution of serum metabolites in COVID-19-positive and COVID-19-recovered patients. Serum samples from COVID-19-positive (CoV) and COVID-19-recovered (Rec) patients were obtained, and the serum metabolites were quantitated using ultra-performance liquid chromatography–mass spectrometry (UPLC-MS). The differential expression of serum metabolites among CoV and Rec patients was represented as a volcano plot of –log10 (*p*-value) (Y-axis) versus log2 (Fold Change) (X-axis) for (**a**) positive and (**b**) negative ion modes of UPLC-MS spectra.

Volcano plots were generated to show the pairwise comparisons of the differential metabolite levels in serum samples from COVID-19-positive and COVID-19-recovered subject groups in positive and negative ion modes (Figure 3). −log_10_ of p-value was plotted on Y-axis, while log_2_ fold change was plotted on X-axis to generate the volcano plot. Fold changes greater than ±1 on the logarithmic (base2) scale were significant. 

**Figure 4:**
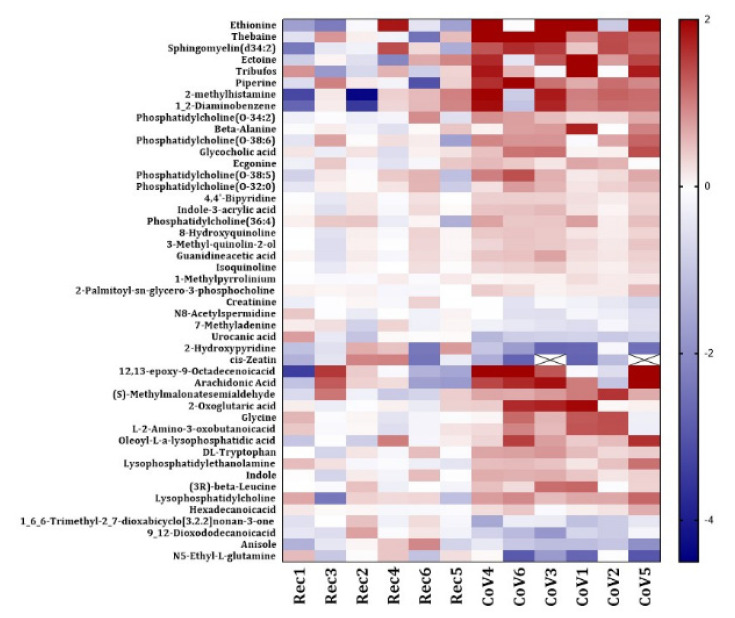
Heat map showing differential levels of serum metabolites in COVID-19-positive and COVID-19-recovered patients. The fold changes of the normalized abundances of various metabolites found in the serum samples from COVID-19-positive (CoV) and COVID-19-recovered (Rec) patients were calculated and the metabolite levels that were significantly altered were represented as a heatmap. The fold changes are color coded in the heat map, with red representing a positive fold change (increase) and blue representing a negative fold change (decrease). For several samples, the fold change values were below the ranges specified in the heat map. These values are represented with a cross (X-mark).

We generated a heat map for significantly altered metabolites found in COVID-19-positive subjects as compared to COVID-19-recovered group. We identified 47 differentially altered metabolites in the serum samples from COVID-19-positive patients in our study (Figure 4). Each metabolite is depicted in an individual row of the heat map while the color scale represents the relative fold changes. A detailed information about the altered miRNAs with their respective *p*-values has been listed in Tables S1 and S2. 

### 2.3. Dysregulated Lipid Metabolism in Patients with COVID-19

**Figure 5:**
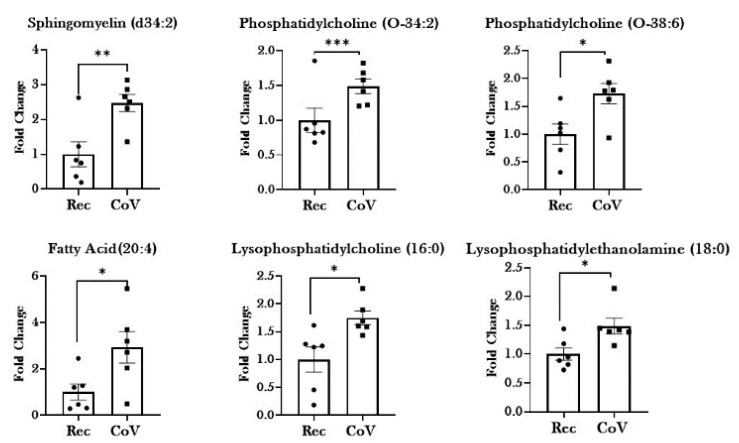
Dysregulation of lipid metabolism in patients with COVID-19. Metabolomic analyses of serum samples from COVID-19-positive (CoV) and COVID-19-recovered (Rec) patients were performed using UPLC-MS. Fold changes in the levels of serum lipids and their metabolites in CoV and Rec samples were plotted as a bar graph. Data are shown as mean ± SEM (*n* = 6/group). * *p* < 0.05, ** *p* < 0.01, *** *p* < 0.001 vs. Rec as per unpaired *t*-test.

Evidence from the literature suggests that the changes in lipid profiles occur during infection as a part of the innate immune response [15]. Our results also show significant changes in the metabolites from lipid metabolism in COVID-19-positive patients. We observed significant increase in the levels of serum lipids including: SM (d34:2), PC (O-34:2), PC (O-38:6), PC (O-32:0), PC(O-38:5), PC(O-36:4), PC (16:0), FA (20:4), LPC (16:0), LPE (18:0), Oleoyl-L-α-lysophosphatidic acid, and Hexadecanoic acid in COVID-19-positive patients as compared to COVID-19-recovered subjects. Conversely, the levels of 9,12-Dioxododecanoic acid were found to be significantly lowered in patients with COVID-19 as compared to recovered individuals (Figure 5 and Table 1).

### 2.4. Significant Alterations in Products from Tryptophan Metabolism in Patients with COVID-19

**Figure 6:**
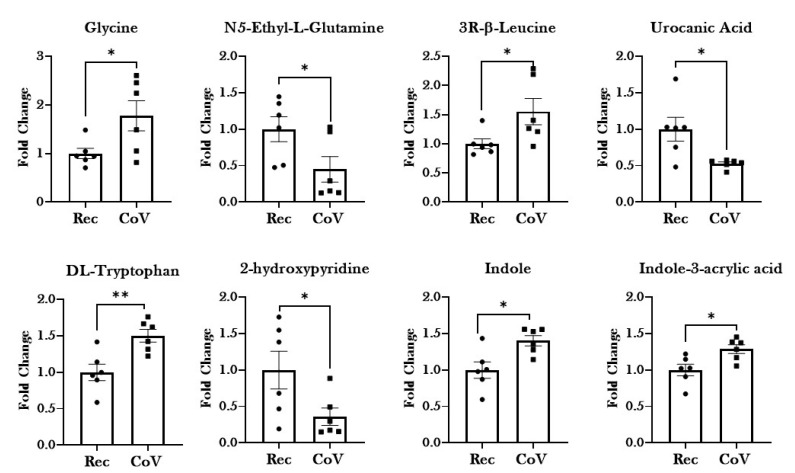
Dysregulation of amino acid metabolism in patients with COVID-19. Metabolomic analyses of serum samples from COVID-19-positive (CoV) and COVID-19-recovered (Rec) patients were performed using UPLC-MS. Fold changes in the levels of serum amino acids and their metabolites in CoV and Rec samples are plotted as a bar graph. Data are shown as mean ± SEM (*n* = 6/group). * *p* < 0.05, ** *p* < 0.01, vs. Rec as per unpaired *t*-test.

Significant increases in the levels of essential and non-essential amino acids—glycine, leucine and tryptophan—were reported in the COVID-19-positive patient group as compared to COVID-19-recovered individuals. Importantly, pronounced dysregulation was observed in the serum levels of products of tryptophan metabolism, including Indole, 2-hydroxypyridine and Indole-3-acrylic acid among the COVID-19-positive patient group (Figure 6). Additionally, dysregulation in the products of amino acid metabolism (Guanidine acetic acid, L-2-amino-3-oxobutanoicacid, (S)-Methylmalonate-semialdehyde, N5-Ethyl-L-glutamine and Urocanic acid) was observed in the serum from COVID-19 patients, thus suggesting a potential role of this metabolic pathway in the pathogenesis of the disease.

### 2.5. Other Dysregulated Metabolites in the Serum from COVID-19-Positive Patients

In addition to the above-mentioned metabolites, we identified dysregulation in various other metabolites in the serum from COVID-19-positive patients as compared to COVID-19-recovered individuals. Of these, most of the metabolites were products of drug metabolism. These included metabolites like thebaine, 1,2-diaminobenzene, ecgonine, 4,4’-bipyridine, 8-hydroxyquinoline, 1-methylpyrrolinium, 3-Methyl-quinolin-2-ol, Isoquinoline, and 1,6,6-Trimethyl-2,7-dioxabicyclo [3.2.2] nonan-3-one. Except for 1,6,6-Trimethyl-2,7-dioxabicyclo [3.2.2] nonan-3-one, all of the drug metabolites were higher among COVID-19-positive patients, thus suggesting that these were the byproducts of the administered treatment for COVID-19 (Table 1). 

Serum levels of other metabolites like ectoine, 2-methylhistamine, glycocholic acid, and 2-oxoglutaric acid was found to significantly higher in COVID-19-positive patients, while that of serum creatinine and cis-zeatin was significantly decreased (Table 1).

## 3. Discussion

The present study aims to compare the metabolomic serum profiles of COVID-19-positive patients and COVID-19-recovered subject groups to identify the potential pathways and metabolites that are involved in the disease pathogenesis of SARS-CoV2 infection. Dysregulated metabolic pathways have been associated with various disorders including cardiovascular, neurological, renal and infectious diseases [16,17,18,19]. In this respect, several studies have been conducted that look at the metabolic profiles of COVID-19-positive patients and healthy subjects [3,8,20]. However, there is a significant gap in knowledge regarding the metabolic changes in COVID-19-positive and COVID-19-recovered subject groups to understand the pathophysiology of the disease and its recovery in humans. To this end, we employed UPLC-MS to study the metabolic profiles of the metabolites in the blood serum from COVID-19-positive patients and COVID-19-recovered subject groups. 

Our investigations identified significant upregulation in the metabolites involved in lipid metabolism in the blood from COVID-19-positive patients as compared to recovered subjects. Previous reports have shown the involvement of cholesterol, sphingomyelins (SM) and phosphatidylcholines (PC) in the formation of the immunological synapse, macrophage activation, NK cell function, and differentiation and activity of T and B effector cells [21,22,23,24]. In accordance with this, we also show comparatively higher levels of sphingomyelins and phosphatidylcholine in the serum from COVID-19-positive patients, which was lowered on recovery. Arshad et al. (2019) demonstrated that lowered phosphatidylcholine levels and increased sphingomyelinase activity are associated with community-based pneumonia [21]. By comparing the levels of phospholipids from this study with another cohort from our lab comprising of healthy non-smokers, we found significant decline in the levels of sphingomyelin and phosphatidylcholine in diseased individuals as compared to healthy subjects (data not shown). Importantly, the levels of these phospholipids declined drastically even on recovery, thus pointing towards prolonged dysregulation in the serum phospholipids on COVID-19 infection. Taken together, our study shows that sphingolipid and phosphatidylcholine metabolism have important roles in regulating SARS-CoV2 infection that could develop as a target for therapeutic interventions for COVID-19 [9,25].

Our results further show increase in the levels of arachidonic acid [FA (20:4)] in the serum of COVID-19-positive patients. Literature suggests that arachidonic acid pathway is involved in the modulation of various inflammatory responses and their resolution [26,27]. In fact, arachidonic acid metabolism is associated with production of cytokines and in cytokine storm in cases of severe COVID-19 [28,29,30]. The results of our current work substantiate these previous findings by confirming the dysregulation in levels of arachidonic acid on SARS-CoV2 infection. Evidence suggests that perturbations in arachidonic acid pathways could lead to an imbalance between the pro-inflammatory metabolites of arachidonic acid including mid-chain hydroxyeicosatetraenoic acid (HETEs) and terminal HETE (20-HETE) [31]. Previous work by our group demonstrated increased levels of 15-HETE and 20-HETE in the serum of COVID-19-positive patients [32]. The elevated levels of arachidonic acid in the patient sera from COVID-19-positive individual further points towards the crucial nature of this metabolic pathway in the pathophysiology of COVID-19.

We also show dysregulation in amino acid metabolism on SARS-CoV2 infection in our study. Amino acid levels are important for the cross talk between host–pathogen interactions. Amino acid metabolism is required to support host defenses by (a) regulating activation of immune cells, (b) maintaining cellular redox state and (c) modulating antibody and cytokine-mediated responses against pathogens. On the other hand, pathogens hijack the host’s amino acid metabolism for its own advantage. Amino acids like arginine, asparagine, and tryptophan, are crucial for both host and pathogen who compete for their availability [33,34,35]. Our investigations reveal dysregulated levels of metabolites of glycine, arginine, glutamine and histidine metabolism on COVID-19 infection. Importantly, we found a significant increase in the level of tryptophan in the serum of COVID-19-positive patients. In fact, the levels of indole and indole-3-acrylic acid (products of tryptophan metabolism) were found to be significantly elevated among COVID-19-positive patients as compared to COVID-19-recovered individuals. No change was observed in the levels of kynurenines in our study. This is contrary to the currently available literature, which points towards lowered tryptophan serum levels on infection [36,37,38].

Recent evidences suggests a close association between infection and tryptophan metabolism [5,39,40]. Tryptophan catabolism is linked to infection-induced inflammation in several infections including *Chlamydia psittaci*, herpes simplex virus (HSV)-2, *Leishmania donovani*, and *Toxoplasma gondii* [39,41,42,43,44]. In fact, due to its significance in orchestrating the immune responses in event of an infection, changes in tryptophan metabolism have been studied in people infected with SARS-CoV-2 [11,20,36,37]. Metabolomic study conducted by Thomas et al. concurred with our findings and showed tryptophan metabolism to be the leading pathway affected by SARS-CoV2 infection. However, contrary to our findings, they found decreased levels of tryptophan and increased levels of the products of tryptophan metabolism (i.e., kynurenine, kynurenic acid, picolinic acid, and nicotinic acid) in the plasma of COVID-19-positive patients [20]. Another study compared the ratio of Kynurenine: tryptophan (Kyn:Trp) among SARS-CoV2-positive and -negative individuals and found a pronounced increase in the Kyn:Trp in the SARS-CoV-2-positive group [37]. All these studies prove the importance of tryptophan metabolism in the disease pathogenesis of COVID-19. 

Importantly, we found increased tryptophan levels in the serum from COVID-19-positive patients as compared to COVID-19-recovered individuals. This is contrary to what was expected. It is important to mention here that all the previously cited studies showing lowered tryptophan concentrations compared COVID-19 patients’ plasma metabolites with healthy individuals, whereas we compared COVID-19-positive patient group with COVID-19-recovered population. Nevertheless, the implications of our results and other studies are important to deduce. There could be several explanations for our observations. First, this could mean that dysregulated tryptophan catabolism continues for days after recovery and could explain the prolonged symptoms of fatigue, weakness and attention deficit in the COVID-19-recovered patient population. Since we did not compare the tryptophan levels in COVID-19-positive and COVID-19-negative patient populations, this speculation needs to be tested in the future. In the event of prolonged dysregulation in tryptophan metabolism among COVID-19-recovered patients, tryptophan supplementation might improve the quality of life during recovery among COVID-19 patients (a subject not being currently investigated/discussed). Second, it is possible that considering the importance of tryptophan metabolism in modulating the immune responses against infectious pathogens, the patients with COVID-19 included in this study received tryptophan supplementation as a medication. Since we currently do not have a detailed account of the medications being administered to these patients, this is a possibility that needs to be reviewed. Third, it is possible that the tryptophan levels and the route of its metabolism are affected by the stage and severity of infection. Essentially, there are two routes of tryptophan metabolism: Kynurenine pathway and the Indole-3-propionic acid (I3P) pathway. Both of these pathways result in activation of AhR leading to suppression of immune response (via exhaustion of NK cells and CD8+ T cells) [38]. Various studies have tested the kynurenine: tryptophan ratio to determine its effect on the progress of infection, but the activation of kynurenine-independent pathway has not been tested [36,37,45]. Nevertheless, our results provide evidence of I3P activation, as indicated by increased circulating levels of Indole-3-acrylic acid. Furthermore, it is possible that there exists a stage- and severity-dependent switch in the regulation of tryptophan metabolism during COVID-19 infection. In this respect, Danlos et al. (2021) performed a stage-dependent metabolomic study in COVID-19 patients and identified 77 metabolites (including products of kynurenine pathway) that were altered in critically ill COVID-19 patient plasma as compared to milder cases [3]. This proves the importance of correlating the results from ‘omic’ studies with disease state and severity for better interpretation. Since this is a preliminary study, it did not allow us the time or resources to gain access to information like disease severity and the stage of each patient included in this work. However, the results from this work will play a pivotal role in designing our future experiments, where we intend to include detailed patient information and provide a stage-dependent account of changes in patient responses.

We also reported increased levels of various different drug metabolites in the serum of COVID-19 patients, including thebaine (opioid drug metabolite) [46], ecgonine (cocaine metabolite) [47], 4,4’-bipyridine (metabolite of heart failure drug) [48], and 8-hydroxuquinoline (anti-fungal drug) [49]. This is not surprising, as many of these drugs could be administered to the patients to manage the disease symptoms. Though we intended to compare the metabolite profiles based on the smoking histories of the two patient populations, we did not observe any noticeable change in the metabolite profiles of smokers versus non-smokers in this study.

Overall, our study provides evidence for dysregulated phospholipid and tryptophan metabolism in patients with COVID-19 infection. However, this study suffers from the limitations of a small sample size, lack of COVID-19-negative controls, and non-availability of detailed patient information describing previous disease history along with the drugs administered during treatment. To overcome the limitation of healthy controls, we used the metabolomic data from healthy non-smoking controls from an existing cohort to compare with this study [50]. These investigations also reveal significant dysregulation in phospholipid and tryptophan metabolism in COVID-19-positive patients as compared to healthy individuals. While we understand that future work with a larger sample size and inclusion of healthy controls in important to get a better understanding of the disease pathogenesis, we cannot undermine the importance of this work that shows the relevance of studying metabolic changes in the serum of COVID-19 patients even after recovery. 

In conclusion, we show dysregulation in phospholipid and tryptophan metabolism in patients with SARS-CoV2 infections as compared to the individuals who recovered from COVID-19. Future research is warranted to determine the relevance of lipid and tryptophan metabolism at every stage of COVID-19 infection to enable identification of effective biomarkers and designing better therapeutic interventions in case of SARS-CoV2 infection.

## 4. Methods

### 4.1. Ethics and Approvals

All the procedures performed in this study were in accordance with the protocols approved by the Institutional Biosafety Committee at the University of Rochester Medical Center, Rochester, NY (Approval number: Rahman/102054/09-167/07-186). The COVID-19-positive (CoV) and COVID-19-recovered (Rec) patient serum was obtained from a commercial provider—BioIVT (Westbury, NY, USA). BSL2+ level of containment for Clinical and Research Safety were followed to perform all the laboratory procedures employed in this study.

### 4.2. Human Subjects

We obtained patient sera from COVID-19-positive (CoV) and COVID-19-recovered (Rec) subjects from BioIVT (Westbury, NY, USA) [32]. The de-identified samples did not require any local IRB requirements or consent forms under 45 CFR 46.102. The samples included in this study were collected between April–May 2020. Antibody (Diazyme serological assay) test for performed by BioIVT to confirm the COVID-19 infectivity for both CoV and Rec patient samples. Per CDC guidelines, the COVID-19-positive patients who were convalescent 30 days post appearance of their last symptom (fever, cough, shortness of breath, etc.) were classified as ‘COVID-19-recovered’. For the purposes of this study, both current and ex-smokers were classified as ‘Smoker’ group, while patients who had never smoked were termed as ‘Non-smoker’. A detailed description of the clinical characteristics of the study subjects is given in Table 2.

### 4.3. Metabolomic Analyses

Metabolomic analyses were performed using ultrahigh-performance liquid chromatography/tandem mass spectrometry (UHLC/MS/MS) at the Mass Spectrometry Core at Georgia Institute of Technology, as described earlier [50,51]. The major components of the process are discussed below:

#### 4.3.1. Nomenclature 

The abbreviations used for various classes of metabolites included in the study are as follows: sphingomyelin, SM; phosphatidylcholines, PC; phosphatidylethanolamine, PE; fatty acid, FA; lysophosphatidylcholines, LPC; lysophosphatidylethanolamine, LPE; and triacylglycerol, TG.

#### 4.3.2. Sample Preparation

Serum samples of 200 µL mixed with HPLC-grade methanol (1:1 ratio) were shipped on dry ice to Georgia Institute of Technology. These samples were thawed, vortexed and centrifuged at 21,100 g for 5 min to remove any debris. A quality control check was performed by combining 20 µL from each sample being tested. The remaining supernatant was transferred to LC vials and stored at 4 °C until further use. Additionally, 80% methanol was used as blank for this study.

#### 4.3.3. LC/MS Data Acquisition

Hydrophilic interaction liquid chromatography (HILIC) was used for metabolite separation using ACQUITY UPLC HILIC systems (Waters Corporation, Milford, MA, USA). A BEH amide column (150 × 2.1 mm) with a particle size of 1.7 µm was used for separation. Water/acetonitrile (80:20) solution with 10mM ammonium formate and 0.1% formic acid served as mobile phase A, whereas mobile phase B constituted of 100% acetonitrile with 0.1% formic acid. Metabolites were analyzed using liquid chromatography coupled with tandem Orbitrap/Ion trap mass spectrometry. Samples were analyzed in both positive and negative mode with m/z between the ranges of 50 and 1500. 

#### 4.3.4. Data Analyses

Data analyses for the identified metabolite data were performed using a data processing method described previously [50,51]. In brief, Compound Discoverer v2.1 (ThermoFisher Scientific, Waltham, MA, USA) software was used for metabolite identification, chromatographic alignment and metabolite quantification. Mass spectral ion adduct analysis was first performed to ensure unambiguous metabolite assignment in each mass spectrum. Elemental formulas were then generated on the basis of exact masses with an error of ±10 mDa and isotopic patterns. Tentative identities were searched against the human metabolome database (HMDB) [52], the Metlin [53] database, and the Lipid Maps [54] database. Tandem MS databases such as Metlin, MassBank [55] and mzCloud were further used together with literature searches to confirm the metabolite identity. 

The area under mass spectra for each metabolite was used to calculate the fold changes. The average of the normalized area from COVID-19-recovered samples was used as baseline control for this study. The fold changes for metabolites with a *p*-value below or equal to 0.05 were shortlisted and used to plot the heat map.

### 4.4. Statistical Analyses

GraphPad Prism 8.0 software was used for all statistical analyses. Two-tailed unpaired *t*-test was employed to determine significant changes between the diseased samples and controls. Data were presented as mean + SEM and a *p*-value < 0.05 was considered statistically significant.

## Figures and Tables

**Table 1:** Significantly altered metabolites in the serum of COVID-19-positive patients with observed fold changes.

Name	Molecular Weight	Log2 Fold Change: (Co)/(Re)	*p*-Value: (Co)/(Re)
Lipid and Lipid metabolites			
SM(d34:2)	700.55	1.70	0.02
PC(O-34:2)	743.58	0.82	0.03
PC(O-38:6)	791.58	0.74	0.05
PC(O-32:0)	719.58	0.39	0.06
PC(O-38:5)	793.60	0.53	0.04
PC(O-36:4)	767.58	0.96	0.08
PC(16:0 > PC(0:0/16:0)	495.33	0.19	0.01
FA(20:4)	304.24	1.82	0.04
Oleoyl-L-α-lysophosphatidic acid	436.26	0.76	0.02
Hexadecanoicacid	256.24	0.25	0.05
1_6_6-Trimethyl-2_7-dioxabicyclo[3.2.2]nonan-3-one	184.11	−0.56	0.02
9_12-Dioxododecanoicacid	228.14	−0.70	0.01
LPC(16:0) > LPC(16:0/0:0)_and_LPC(0:0/16:0) 2M + H2CO2	541.34	0.42	0.08
LPE(18:0) > LPE(18:0/0:0)_and_LPE(0:0/18:0)	481.32	0.61	0.01
Amino Acid and Amino acid metabolites			
DL-Tryptophan	204.09	0.64	0.01
Indole;1-Benzazole	117.06	0.53	0.03
2-Hydroxypyridine	95.04	−2.13	0.05
Indole-3-acrylic acid	187.06	0.37	0.03
Glycine	75.03	0.98	0.05
Guanidineacetic acid	117.06	0.33	0.02
L-2-Amino-3-oxobutanoicacid	117.04	0.79	0.04
(3R)-beta-Leucine	131.09	0.47	0.04
(S)-Methylmalonatesemialdehyde	102.03	1.17	0.02
N5-Ethyl-L-glutamine	174.10	−2.32	0.04
Urocanic acid	138.04	−0.89	0.02
Drug Metabolites			
Thebaine	311.15	1.87	0.01
1_2-Diaminobenzene	108.07	0.89	0.10
Ecgonine	185.11	0.64	0.04
4,4’-Bipyridine	174.08	0.35	0.02
8-Hydroxyquinoline	145.05	0.36	0.03
1-Methylpyrrolinium	83.07	0.21	0.03
3-Methyl-quinolin-2-ol	159.07	0.35	0.03
Isoquinoline	129.06	0.31	0.02
Others			
Ectoine	142.07	1.59	0.03
cis-Zeatin	219.11	−2.10	0.05
2-methylhistamine	125.10	0.91	0.10
Glycocholic acid	465.32	0.66	0.02
Creatinine	113.06	−0.31	0.04
2-Oxoglutaric acid	146.02	1.13	0.04

**Table 2:** Clinical characteristics of COVID-19-positive and COVID-19-recovered patients used in this study.

Characteristics	COVID-19-Recovered	COVID-19-Positive	*p*-Value *
N	6	6	
Age (mean + SD)	36.33 ± 9.4	47 ± 14.4	0.1926
Male:Female	3:3	4:2	0.6667
Smokers, n (%)	2 (33.33)	2 (33.33)	0.3333

* Mann–Whitney’s test.

## Data Availability

The data are presented in the manuscript and hence are publicly available, but the clinical data are not publicly available due to privacy and ethical reasons.

## Supplementary Materials

The following are available online at https://www.mdpi.com/article/10.3390/metabo11100659/s1, Table S1: Fold changes of metabolites detected under positive mode in serum from COVID-19 positive and COVID-19 recovered subjects, Table S2: Fold changes of metabolites detected under negative mode in serum from COVID-19 positive and COVID-19 recovered subjects.

## List of Abbreviations

COVID-19	Coronavirus Disease-2019
SARS-CoV2	Severe Acute Respiratory Syndrome Coronavirus 2
WHO	World Health Organization
UPLC-MS	Ultrahigh Performance Liquid Chromatography/tandem mass spectrometry
HIV	Human Immunodeficiency Virus
AhR	Aryl hydrocarbon Receptor
I3P	Indole-3-propionic acid
CD	Cluster of differentiation
