# Supplementary material for: SARS-CoV2 Infection Alters Tryptophan Catabolism and Phospholipid Metabolism
Source: Metabolites. 2021 Sep 28;11(10):659. doi: 10.3390/metabo11100659 (PMC8538244; doi:10.3390/metabo11100659)
Supplement: Supplementary file 1 [file metabolites-11-00659-s001.zip › metabolites-1369346-SI.pdf]

Supplementary Files

SARS-CoV2 infection alters Tryptophan catabolism and phospholipid metabolism

Gagandeep Kaur[1], Xiangming Ji[2] and Irfan Rahman[1*]

Table S1. Fold change of the metabolites detected under the positive mode in serum from COVID-19 positive and COVID-19 recovered subjects.

Name	Log2(FC) Rec01	Log2(FC) Rec02	Log2(FC) Rec03	Log2(FC) Rec04	Log2(FC) Rec05	Log2(FC) Rec06	Log2(FC) Cov01	Log2(FC) Cov02	Log2(FC) Cov03	Log2(FC) Cov04	Log2(FC) Cov05	Log2(FC) Cov06
**Ethionine**	-1.692645561	-0.117905234	-2.325363341	1.82079704	-1.64170057	-0.481264696	5.21911835	-0.933324439	2.077527589	3.60647961	3.68314624	-0.068909251
**Thebaine**	-0.533806594	0.120575275	0.816375872	-0.234642274	0.519844043	-2.494678282	0.899550532	1.389684927	2.156221728	2.156010699	1.243770155	3.219002269
**SM(d34:2)**	-2.393295169	-0.259732471	-0.410458003	1.394017353	-1.449037241	0.295917728	0.442020047	1.41164346	1.518981797	1.326858152	1.213198644	1.648402464
**Ectoine**	-0.862557249	-0.572892842	0.079566208	-2.134736494	0.941650483	0.653171495	2.315898654	0.759840405	1.304108955	1.669838273	1.456934597	-0.463496656
**Tribufos**	0.839767242	-0.69489244	-1.815797681	0.589342218	0.329835426	-0.870372683	2.042698775	-0.05489084	-0.155038664	1.811768826	1.786675637	0.567100255
**Piperine**	-0.616198345	0.160424438	0.976108897	-0.215217521	0.359299534	-3.075528819	0.644466733	1.136151096	1.105440465	1.616012445	0.931055725	2.898625031
**2-methylhistamine**	-3.23531784	-4.390009584	0.176971561	0.365184017	0.98098911	0.539773659	1.000981444	1.219751067	1.791961712	1.971625893	1.141612552	-0.916175461
**1_2-Diaminobenzene**	-2.733814757	-3.497797657	0.154420167	0.329317631	0.950829503	0.544754486	0.959625589	1.14332082	1.710097515	1.904457765	1.116742063	-1.059896256
**PC(O-34:2)**	-0.27712537	-0.300506826	-0.068201722	-0.195465518	-0.553031883	0.890715756	0.267278026	0.28653259	0.502480705	0.86628682	0.660982118	0.747411377
**Beta-Alanine**	-0.077582199	-0.201405616	0.14450413	-0.550930211	0.451723131	0.03850469	1.745077662	-0.026942512	0.778649978	0.102914327	0.97479596	0.739388011
**PC(O-38:6)**	-0.476546052	0.031271855	0.717336437	0.252971092	-1.67281773	0.15049225	-0.1002437	0.701098193	0.839107789	0.946412962	1.211035581	0.832523039
**Glycocholic acid**	0.19842062	0.212445536	-0.333307779	-0.612264813	0.222434012	0.110128311	0.08457977	0.111579499	1.109251467	0.48167226	1.38293904	1.087849262
**Ecgonine**	-0.272266022	-0.165433422	0.421636618	-0.529268261	0.430864821	-0.152118644	0.691444904	0.581319285	0.19551077	0.538467135	0.025401498	0.417709837
**PC(O-38:5)**	-0.826630882	0.029369113	0.177544752	0.410483351	-1.132421115	0.584338253	0.169223279	0.278724908	0.611791857	0.997351377	0.668274368	1.379398766
**Leucine**	-0.005573512	0.143505432	-0.109417752	-0.242729285	-0.077345393	0.23800845	0.566845407	0.12362031	0.520934176	0.381821781	0.597200001	-0.070029718
**PC(O-32:0)**	-0.457508042	0.030996135	0.113070904	0.206801535	-0.913396222	0.56895878	0.206794629	0.353875183	0.568975507	0.241268284	0.555944336	0.775155302
**DL-Tryptophan**	0.041589825	0.202436743	-0.580014722	-0.137930367	0.031815962	0.286923145	0.218423988	0.079621489	0.548037998	0.460274504	0.366280273	0.475740169
**4,4'-Bipyridine**	-0.042017264	0.188774084	-0.408029731	-0.070729543	0.005829721	0.237627562	0.258338001	0.150939878	0.362884577	0.376419129	0.335288085	0.372029465
**Indole-3-acrylic acid**	0.039262394	0.198087973	-0.574150368	-0.137114045	0.032681094	0.288426634	0.224812816	0.079432536	0.538799135	0.459329811	0.360285051	0.471169107
**PC(36:4)**	0.112805709	0.443772669	0.41920788	-0.337215227	-1.424902864	0.080397077	0.751618349	0.14413477	0.438290606	0.717200331	0.49507869	0.428284587
**8-Hydroxyquinoline**	-0.033456698	0.133111908	-0.54687233	-0.110384277	0.067626942	0.341907794	0.208613249	0.139625792	0.415754191	0.420732223	0.333679733	0.460775849
**4,4'-Bipyridine**	-0.048177786	0.182324995	-0.363351688	-0.048870322	-0.011289492	0.216456897	0.226652306	0.111230492	0.2973553	0.365564232	0.351372682	0.355877526
**3-Methyl-quinolin-2-ol**	-0.008666793	0.113201427	-0.60076219	-0.043717791	0.058205186	0.326118574	0.3146586	0.179092025	0.420139211	0.328131982	0.446355826	0.451174074

**Guanidineacetic acid**	0.066523154	0.154476756	-0.590268518	-0.168349716	0.084478866	0.293262308	0.269813219	0.181193236	0.714095763	0.453518362	0.365823412	0.480954506
**Isoquinoline**	-0.032184761	0.081864505	-0.392097005	-0.015387281	0.026138376	0.255778632	0.199574608	0.122390052	0.417078213	0.326877701	0.306448924	0.361176087
**1-Methylpyrrolinium**	-0.042327814	-0.09560824	-0.109211094	0.145586672	0.163075228	-0.089131221	0.266077117	0.161688424	0.111040231	0.069412194	0.186254475	0.117023515
**PC(16:0>PC(0:0/16:0)**	0.114614126	0.108835416	0.071566767	-0.162096495	-0.003705551	-0.156916971	0.16331689	0.15784376	0.09676861	0.391072706	0.539352632	0.277941717
**Creatinine**	-0.301016705	0.064756589	-0.042235878	-0.140889937	-0.016943415	0.351604959	-0.262060142	-0.414421591	-0.135856609	-0.023934678	-0.790781013	-0.521842633
**N8-Acetylspermidine**	0.42975132	-0.358599274	-0.036188874	0.002018702	0.089570399	-0.264658101	-0.183305791	-0.301692129	-0.567584013	-0.026523903	-0.583841645	-0.551573551
**7-Methyladenine**	0.143622863	-0.827326599	0.258140485	0.332502355	0.066859952	-0.270232209	-0.612683305	-0.175284952	-0.522981912	-0.254197447	-0.580339134	-0.403619557
**Urocanic acid**	0.75721489	-1.052095285	-0.405734932	0.041200106	0.037932311	0.024384826	-0.795169919	-0.956007607	-0.834308911	-1.283924986	-0.828246914	-0.888972056
**2-Hydroxypyridine**	-1.095965806	0.630559051	-0.555014803	0.46877027	0.788805517	-2.382403562	-2.67550347	-0.174196611	-2.723501879	-1.018735984	-2.526779161	-1.750955726
**cis-Zeatin**	-1.366762987	0.95894326	-0.466835856	0.984680205	-0.349450644	-2.465567519	-2.742862388	-1.1868165	-6.262792361	-1.444170664	-5.497641683	-2.753046834

Table S2. Fold change of the metabolites detected under the negative mode in serum from COVID-19 positive and COVID-19 recovered subjects.

**Name**	**Log2(FC) Rec01**	**Log2(FC) Rec02**	**Log2(FC) Rec03**	**Log2(FC) Rec04**	**Log2(FC) Rec05**	**Log2(FC) Rec06**	**Log2(FC) Cov01**	**Log2(FC) Cov02**	**Log2(FC) Cov03**	**Log2(FC) Cov04**	**Log2(FC) Cov05**	**Log2(FC) Cov06**
**[FA(18:0)]12R_13S-epoxy-9Z-octadecenoicacid**	-3.406926012	0.401175555	1.55794187	-0.117315247	-1.577505666	-1.381422051	-0.119138251	-0.609885269	1.329924938	2.217315567	2.186346595	1.997371039
**FA(20:4)**	-1.091905656	0.356120769	1.291277262	0.268502851	-1.794047594	-1.687271044	1.024877377	-1.034500135	1.881103456	1.438270859	2.448102245	1.672890937
**(S)-Methylmalonatesemialdehyde**	-0.635333029	-0.248746983	1.07040001	-0.943875721	0.37797752	-0.748523739	1.012137063	1.584341483	0.786060885	0.693124689	0.654486884	0.651153337
**2-Oxoglutaric acid**	0.12938594	-0.047317714	-0.335094912	0.116195952	0.358425927	-0.358812066	1.940562675	0.054427325	1.733931041	0.382849518	0.11014369	1.667802421
**Glycine**	0.568887663	0.064179525	-0.073465237	-0.499491013	-0.084557899	-0.200336531	1.296081006	1.381468526	0.574057841	0.076530738	-0.292063165	1.166338672
**L-2-Amino-3-oxobutanoicacid**	0.429817173	0.051219173	-0.120774038	-0.595713926	0.219335547	-0.198941281	1.326131247	1.375267843	0.540540764	0.285747489	-0.305967592	0.947706377
**Oleoyl-L-α-lysophosphatidic acid**	-1.017144299	-0.865498437	-0.041116117	1.004594859	0.141073614	-0.190928568	0.402018138	0.525062261	0.748181332	0.319706943	1.620690668	1.50312147

**DL-Tryptophan**	-0.000155622	0.192797809	-0.769047263	-0.158658289	-0.063598626	0.503443376	0.510172365	0.290622476	0.817806511	0.695721778	0.397755769	0.73693743
**LPE(18:0)>LPE(18:0/0:0)_and_LPE(0:0/18:0)**	0.524001262	-0.172105403	0.238912621	-0.076369028	-0.460703281	-0.287466299	0.19692674	0.475443551	0.469578226	0.502676401	1.098139609	0.535841482
**Indole;1-Benzazole**	0.010010331	0.152437239	-0.747135814	-0.197136886	-0.030492573	0.520488478	0.433186401	0.194353758	0.641309583	0.638795687	0.353252117	0.610626231
**(3R)-beta-Leucine**	-0.005393254	0.483492711	-0.091442532	-0.290216591	-0.206918168	-0.024943372	1.194563531	-0.063255104	1.132020229	0.490739957	0.337973807	0.275420592
**LPC(16:0)>LPC(16:0/0:0)_and_LPC(0:0/16:0) 2M + H2CO2**	0.689597155	0.354840823	-2.432585998	0.289924535	-1.130519056	0.314712506	0.667949906	0.680803067	0.519319573	0.762918938	1.185887983	0.920233517
**Hexadecanoicacid**	0.178103275	0.061871336	0.043052461	-0.127891221	-0.070629967	-0.109197286	0.129263259	0.216884847	-0.093214719	0.261259286	0.633329263	0.438939084
**1_6_6-Trimethyl-2_7-dioxabicyclo[3.2.2]nonan-3-one**	-0.560765037	0.47220218	-0.01950616	-0.248993401	-0.161599943	0.278277092	-1.114676856	-0.906535302	-0.366421277	-1.54588668	-0.425465673	-0.330891505
**9_12-Dioxododecanoicacid**	-0.42391034	0.731729189	-0.602770692	-0.077167063	-0.238828941	0.18920011	-0.72480731	-0.90389866	-1.836283193	-0.811472009	-0.20612629	-1.23238442
**Anisole**	-1.368158342	0.064960093	-0.44092332	0.431815325	-0.788940575	0.927666014	-1.176681034	-1.022254713	-1.176184064	-0.38995978	-1.96450064	-0.976802453
**N5-Ethyl-L-glutamine**	0.533190632	0.030677438	-0.981158555	0.437453069	0.25664997	-1.071278624	-2.702890921	-0.049742767	-1.785422572	0.041558644	-2.999480952	-2.932790621
